# Role and regulators of N^6^-methyladenosine (m^6^A) RNA methylation in inflammatory subtypes of asthma: a comprehensive review

**DOI:** 10.3389/fphar.2024.1360607

**Published:** 2024-07-23

**Authors:** Ge Gao, Yu Qiu Hao, Chen Wang, Peng Gao

**Affiliations:** ^1^ Department of Respiratory and Critical Care Medicine, The Second Affiliated Hospital of Jilin University, Changchun, China; ^2^ Department of Infectious Diseases, The Second Affiliated Hospital of Jilin University, Changchun, China

**Keywords:** N 6 -methyladenosine, asthma, asthma phenotype, inflammation, inflammatory disease

## Abstract

Asthma is a common chronic inflammatory disease of the lungs and airway, yet its inflammatory subtypes and potential pathogenesis have not been completely elucidated and require further study. With advances in epigenetic development, methylation has emerged as a new direction for identifying and decoding the occurrence and subtype manifestations of asthma. N^6^-methyladenosine (m^6^A), an RNA methylation modification occurring in the N^6^-position of adenosine, is a prevalent epigenetic modification observed in eukaryotes. It exerts significant control over mRNA metabolism by regulating alternative splicing, stability, export, and translation. The dynamic process of m^6^A methylation plays a crucial role in the pathogenesis of asthma and is tightly regulated by three types of regulators: writers, readers, and erasers. This article provides a comprehensive review of the association between m^6^A regulators and the pathogenesis of inflammatory subtypes of asthma, such as involvement of inflammatory cells and related inflammatory response. Furthermore, the findings presented herein provide new insights and a solid foundation for further research on m^6^A mRNA methylation as biomarkers for the diagnosis and development of personalized treatment for different subtypes of asthma, particularly neutrophilic asthma and eosinophilic asthma.

## 1 Introduction

Asthma is a common heterogeneous disease of the respiratory system, and chronic inflammation, allergic reactions, and airway hyperresponsiveness are the main causes of asthma-related morbidity. Despite its prevalence, the inflammatory subtypes and their potential pathogenesis of asthma have not yet been fully elucidated. Hence, we aimed to review previous studies in order to highlight key findings and discover new insights that will guide future research in these areas.

Currently, the internationally applied Global Initiative for Asthma guidelines categorize patients with asthma into four inflammatory subtypes according to a cell-based classification of the inflammatory infiltrate in their sputum: eosinophilic asthma (EA), neutrophilic asthma (NA), mixed granulocyte asthma (MA), and oligo-granulocyte asthma (PA) (Simpson et al., 2006). The EA subtype manifests as the secretion of Th2-type cytokines (IL-4, IL-5, IL-13) and the expression of type 2 innate lymphocytes (ILC2), downstream of which a Th2-type immune response occurs, and then IL-4 stimulates the expression of polyfilament proteins in bronchial epithelial cells, which are important genes for the development and progression of EA (Carr et al., 2018; Gao et al., 2021). Treatment with steroids can be effective for asthmatics with a clear diagnosis of EA, but early diagnosis of EA and precise targeting of EA targets is more effective for patients with severe hormonal complications (Carr et al., 2018). The NA subtype is associated with non-Th2 asthma and is primarily driven by neutrophil extracellular traps (NETs) with large numbers of neutrophils infiltrating the blood and sputum (Hudey et al., 2020; Xie et al., 2022). Inflammatory factors such as IL-1β, IL-6, IL-12, and IL-17 play important roles in the development of NA and steroid resistance, and are also associated with the activation of NLRP3 vesicles and regulating the NF-kB inflammatory signaling pathway during inflammation in asthma (Sánchez-Ovando et al., 2021; Williams et al., 2021; Chen et al., 2022; Chang et al., 2023). Relevant markers for a definitive diagnosis of NA are lacking, and NA is resistant to steroid hormone therapy, making conventional asthma medications less effective (Chung, 2016; Mogensen et al., 2020). The MA subtype presents with 80% neutrophils, 10% eosinophils, and worse lung function in the airways. The mechanism underlying the pathogenesis of MA has not been clarified, but it may be associated with neutrophil inflammation and inflammatory factors such as IL-35 and IL-17A (Li et al., 2020; Menson et al., 2020; Hastie et al., 2021). The PA subtype is induced by sputum showing no significant increase in eosinophilic and neutrophilic inflammation in the airways, with an elevated sputum macrophage count; however, a stable consensus on the pathogenesis of PA has not yet been reached (Olgac et al., 2021). Tliba et al. suggested that PA airway obstruction may result from a pulmonary inflammatory mechanism of hypertrophy and dysfunction of airway smooth muscle tissue; however, PA is insensitive to hormonal treatment (Ntontsi et al., 2017; Tliba and Panettieri, 2019). Further research on targets for asthma subtypes is needed to further refine the diagnosis and treatment of each subtype classification.

The most common epigenetic of eukaryotic RNA modifications, m^6^A modifications were first identified in 1974 for their unique distribution in mRNAs (Tang et al., 2021; Desrosiers et al., 1974; Lence et al., 2019), in which m^6^A methylation sites bind reversibly to various proteins, act at the post-transcriptional stage, affect mRNA stability, are involved in almost all biological activities in the human body, and have been strongly implicated in diseases of the lungs, heart, intestines, brain, muscles, and other organs (Tang et al., 2021; Xiong et al., 2021; Wang et al., 2022; Fei et al., 2022). Relying on a series of regulatory factors, m^6^A regulatory factors carry out various functions and roles. The three main types of functional regulators involved in m^6^A methylation are methyltransferase, methylated reading proteins, and demethylation enzymes, which are known as writes, readers, and erases, respectively, and provide a synergistic effect on the progression of inflammation, tumors, and other related diseases (Yang et al., 2018; Zaccara et al., 2019). m^6^A is assembled by a multicomponent methyltransferase complex, which next recognizes the target mRNA in the presence of binding proteins. Upon binding to the target mRNA, the m^6^A modification regulates the transcription process and metabolism of the RNA, including selective splicing, exportation, stability, and further translation (Yang et al., 2018). The presence of demethylase allows the mRNA-bound m^6^A to detach at the right time (Jia et al., 2011; Zheng et al., 2013). There are 27 widely recognized regulatory factors, including writes: cbl proto-oncogene like 1 (CBLL1), methyltransferase-like 14 (METTL14), methyltransferase-like 3 (METTL3), zinc finger CCCH-type-containing 13 (ZC3H13), the aberrant expression of the zinc finger protein 217 (ZNF217), RNA-binding motif protein 15B (RBM15B), Wilms’ tumor 1-associating protein (WTAP), RNA-binding motif protein 15 (RBM15), and virus-like m6A methyltransferase-associated protein (KIAA1429); readers: YT521-B homology (YTH) domain (YTHDF1, YTHDF2, YTHDF3, YTHDC1, YTHDC2), eukaryotic translation initiation factor 3 subunit A (EIF3A), eukaryotic translation initiation factor 3 subunit B (EIF3B), heterogeneous nuclear ribonucleoprotein A2B1 (HNRNPA2B1), heterogeneous nuclear ribonucleoprotein C (HNRNPC), IGF2 mRNA binding proteins (IGF2BP1/2/3) families, leucine rich pentatricopeptide repeat containing (LRPPRC), fragile X messenger ribonucleoprotein 1 (FMR1), and ELAV-like RNA binding protein 1 (ELAVL1); and erasers: alkB homolog 5 (ALKBH5) and fat mass and obesity-associated protein (FTO) (Yang et al., 2018; Sun et al., 2021; Deng et al., 2022; Mo et al., 2022). According to the expression of 27 m6A regulators, Non‐negative matrix factorization (NMF) was performed to identify three different m6A modification patterns, with FMR1, IGF2BP2, and YTHDC2 accounting for the largest proportion of patterns 1, 2, and 3, respectively. Different patterns play different roles in the infiltration of asthma inflammatory cells, which can be referred to as Modes 1, 2, and 3. Mode one showed higher expression levels of γ-T cells, TH17, macrophages, eosinophils, mast cells, and neutrophils; Mode two affected the infiltration of NK, DC, and monocytes; and Mode three regulated the infiltration of CD8^+^ T cells and TH1 cells in asthma (Sun et al., 2021).

In the presence of regulatory factors, m^6^A is involved in inflammatory and lung diseases such as asthma, respiratory distress syndrome, pneumonia, and lung cancer (Chao et al., 2020; Kim et al., 2021; Li et al., 2021; Fei et al., 2022). In asthma inflammatory subtypes, m^6^A methylation regulates macrophage and T-cell differentiation as well as eosinophil, neutrophil, natural killer (NK), and dendritic cell (DC) infiltration in inflammatory responses to different regulatory factors (Table 1), and regulates inflammatory pathways such as nuclear factor-κB (NF-ΚB) and JAK/STATA (Table 2), which are features used for the classification of asthma inflammatory subtypes (Wang et al., 2019a; Ma et al., 2021; Sun et al., 2021; Cai et al., 2022; Liu et al., 2022).

**TABLE 1:** Regulatory factors and their roles in cellular functions and inflammatory subtypes of asthma.

Regulator	Macrophage		T cells		Neutrophil	Eosinophil	NA	EA	NLRP3	IL-12	IL-6	IL-β	References
	M1	M2	Th1/Th17	Th2									
YTHDF1	**(−)**	**(+)**	**(+)**				**(−)**	**(+)**	**(+)**		**(+)**	**(−)/(+)**	Du et al. (2020), Xing et al. (2021), Li et al. (2022a), Hao et al. (2022), He et al. (2022)
YTHDF2	**(−)**	**(+)**					**(−)**	**(+)**		**(−)**	**(−)**	**(−)**	Huangfu et al. (2020), Kong et al. (2022)
YTHDF3						**(+)**		**(+)**					Sun et al. (2021)
Mettl14	**(+)**	**(+)**	**(−)**				**(+)/(−)**	**(+)**	**(−)**		**(+)**		Du et al. (2020), Lu et al. (2020), Cao et al. (2021), Meng et al. (2022), Zheng et al. (2022), Li et al. (2023)
METTL3	**(+)**	**(−)**	**(+)/(−)**	**(+)**	**(+)**		**(+)**	**(+)**	**(+)**	**(+)**	**(−)/(+)**	**(+)**	Li et al. (2017), Wang et al. (2019a), Wang et al. (2019b), Yang et al. (2021), Zhao et al. (2021), Li et al. (2022b), Kong et al. (2022), Zhou et al. (2022), Zhang et al. (2023a), Wang et al. (2023b), Luo et al. (2023)
FTO	**(+)**	**(+)**	**(+)**	**(−)**			**(+)**	**(+)/(−)**	**(+)**	**(+)**	**(+)/(−)**	**(+)**	Gu et al. (2020), Kim et al. (2021), Yu et al. (2021), He et al. (2023)
ALKBH5			**(+)**		**(+)**		**(+)**						Zhou et al. (2021), Liu et al. (2022), Liu et al. (2023b), Subbarayalu et al. (2023)
WTAP									**(+)**		**(+)**	**(+)**	Lan et al. (2022)
EIF3B						**(−)**		**(−)**					Sun et al. (2021)

**TABLE 2:** Mechanism of action of regulatory factors on signaling pathways.

Regulator	JAK2/STAT3	STAT5	NF-ΚB	STAT1	p38MAPK	p53MAPK	SOCS1	SOCS3	TLR4	References
YTHDF1	**(−)/(+)**		**(+)**				**(+)**	**(+)**	**(+)**	Xing et al. (2021), Li et al. (2022a)
YTHDF2		**(+)**	**(−)**	**(−)**	**(−)**	**(−)**				Yu et al. (2019), Huangfu et al. (2020), Ma et al. (2021), Cai et al. (2022)
Mettl14			**(+)**				**(+)**		**(+)**	Du et al. (2020)
METTL3	**(−)**		**(+)/(−)**				**(+)**	**(+)**	**(+)**	Wang et al. (2019b), Wang et al. (2023b), Luo et al. (2023)
FTO	**(−)**		**(+)/(−)**	**(+)**			**(−)**	**(+)**	**(−)**	Hu et al. (2022), He et al. (2023)
ALKBH5		**(+)**								Liu et al. (2023b)
WTAP			**(+)**							Lan et al. (2022)

**FIGURE 1:**
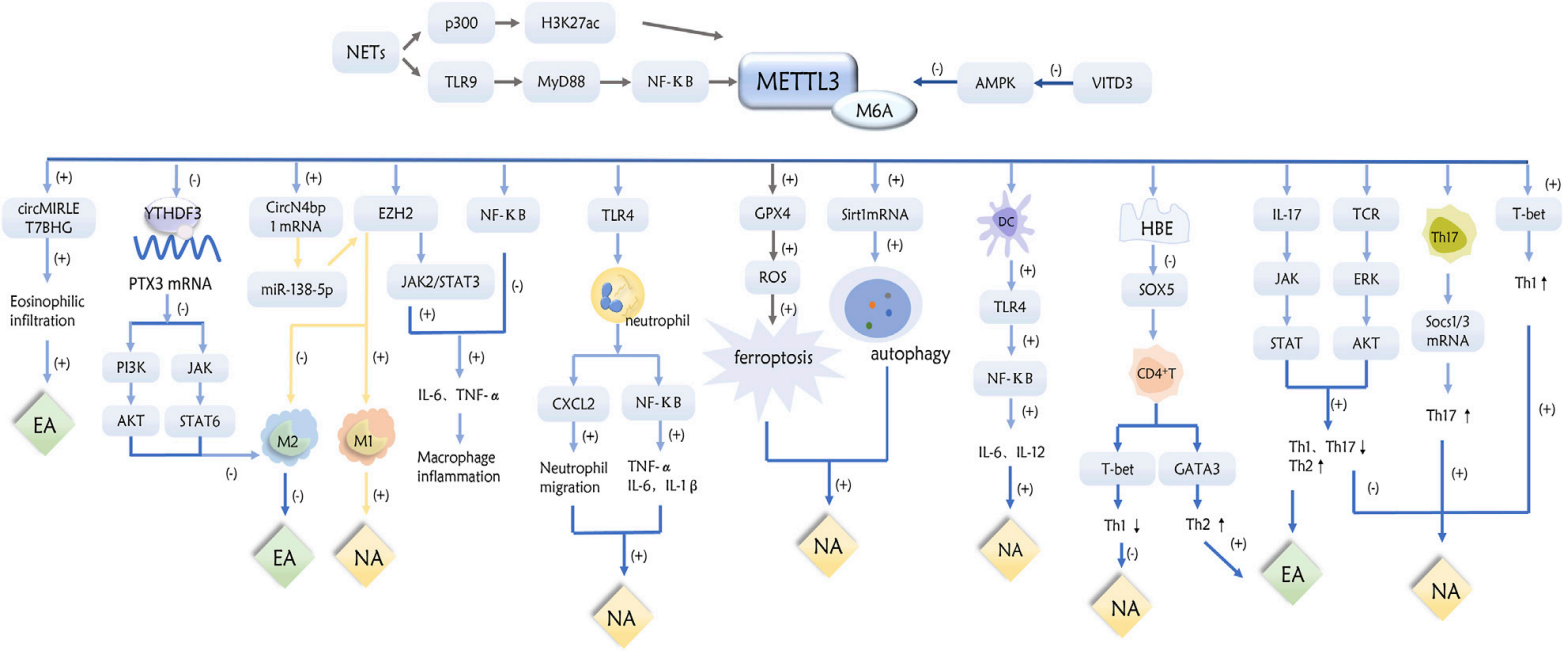
Role of METTL3 in inflammatory subtypes of asthma through a variety of pathways.

**FIGURE 2:**
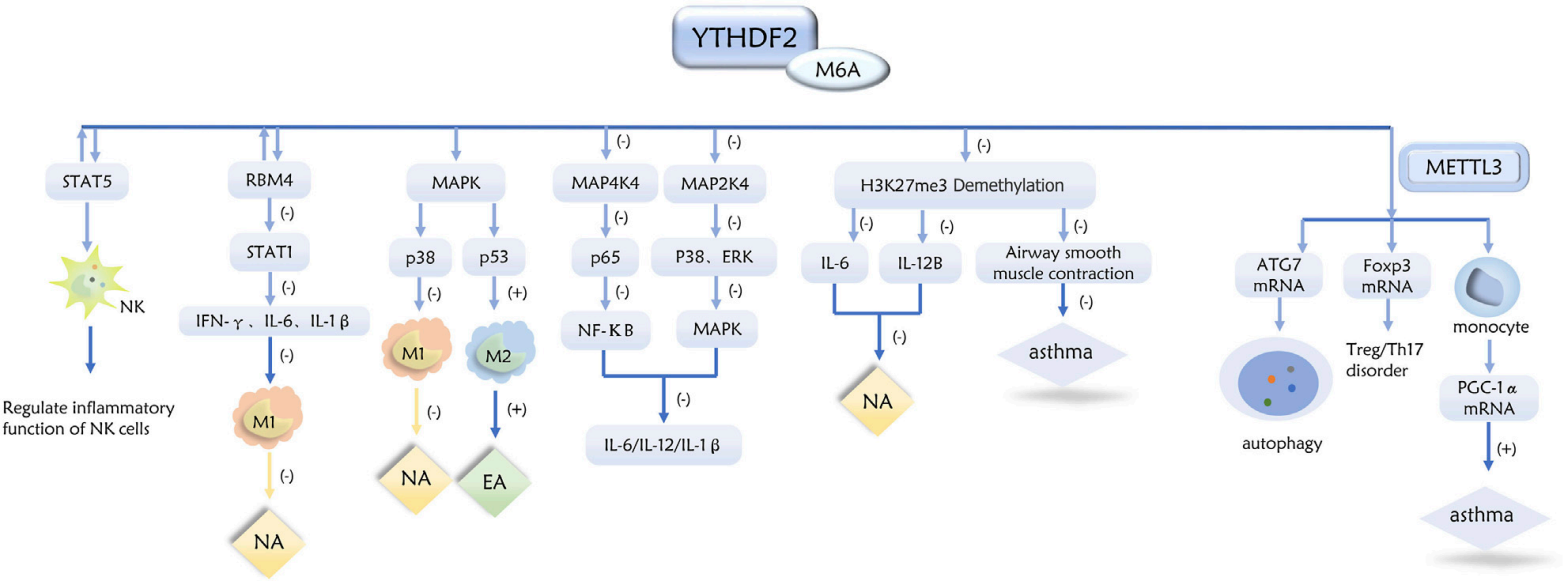
Role of YTHDF2 in inflammatory subtypes of asthma through a variety of pathways.

**FIGURE 3:**
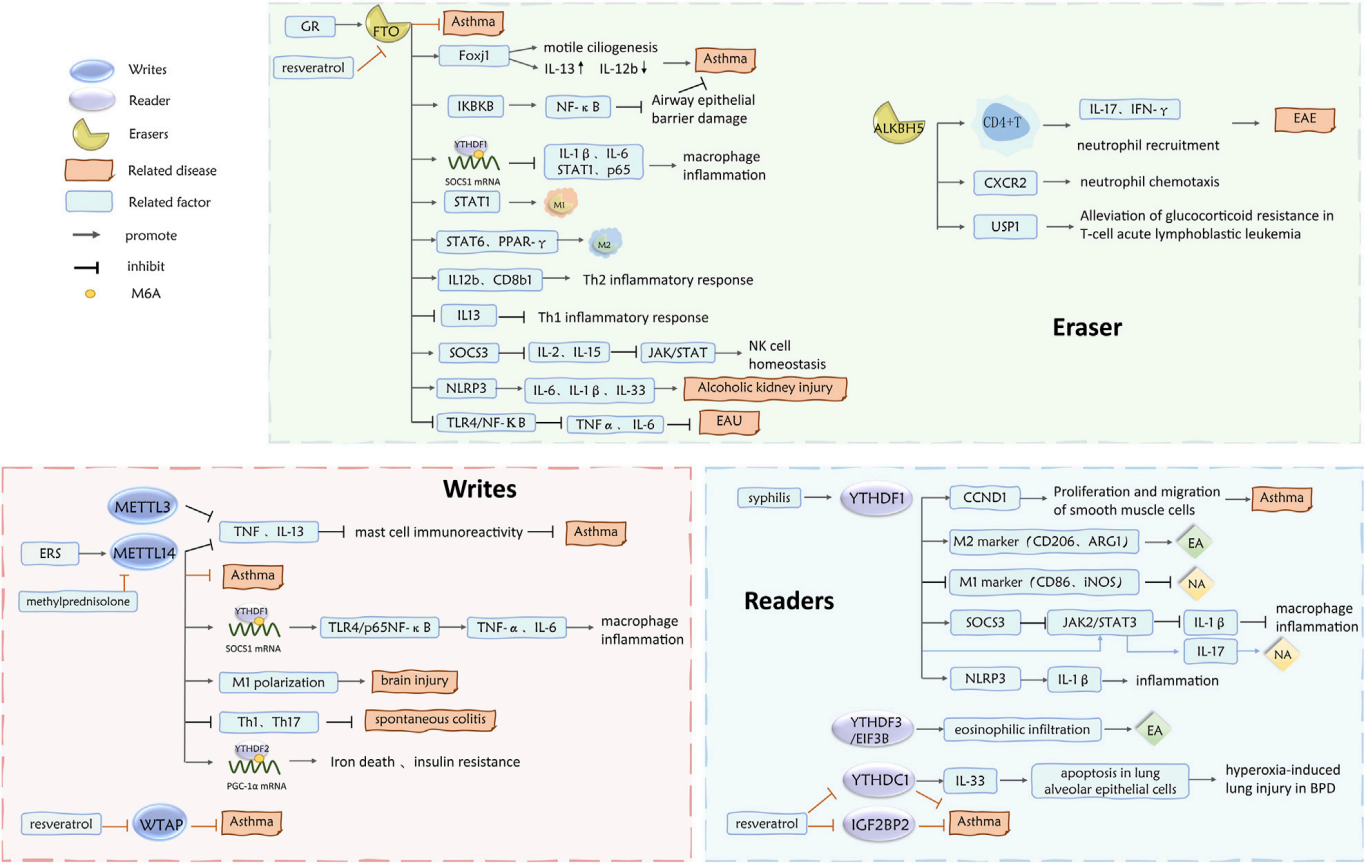
Role of m^6^A writes, readers, and erases in asthma inflammatory subtypes.

This article provides a review of the specific mechanisms by which m^6^A methylation and its associated regulators are involved in the inflammatory response, formation, pathogenesis, and prognosis of asthma and its inflammatory subtypes (Figures 1–3).

## 2 m^6^A RNA methylation: implication in asthma development and progression

m^6^A mRNA methylation is widely involved in various biological functions of the human body, especially in inflammatory and immune responses, and plays an important role in the onset and progression of asthma. By analyzing the dataset of childhood asthma patients obtained from the Gene Expression Omnibus (GEO) database, 11 regulators closely related to asthma were obtained, including: YTHDC1, HNRNPC, YTHDC2, FMR1, YTHDF3, HNRNPA2B1, KIAA1429, METTL3, WTAP, RBM15B, and ZC3H13, of which RBM15B expression was significantly upregulated in asthma, while all other regulators were downregulated (Dai et al., 2021). Further analysis of the above 11 m^6^A regulators using consistent clustering revealed that the expressions of YTHDC1, HNRNPC, FMR1, HNRNPA2B1, KIAA1429, METTL3, RBM15B, and ZC3H13 were associated with Th1 immunity, and the expression of YTHDF3 was associated with Th2 immunity (Xiong et al., 2024). Th1 immunity and Th2 immunity are the immune mechanisms of NA and EA, respectively (Hudey et al., 2020; Gao et al., 2021). A combined analysis of m^6^A methylation and gene expression in asthma lung tissues demonstrated that m^6^A methylation is involved in Th1 cell differentiation and other pathological mechanisms that have been correlated to asthma airway hyperresponsiveness, inflammatory and immune responses in asthma, and asthma inflammatory subtypes, although the specific mechanisms involved need to be further investigated (Teng et al., 2021).

Recent studies have confirmed that FTO, acting as an eraser, downregulated the m^6^A modification on IKBKB mRNA in a mouse asthma model, thereby enhancing its stability and leading to the upregulation of IKBKB expression, which effectively attenuated epithelial barrier damage in PM2.5-exposed cells by regulating NF-κB signaling (Xiong et al., 2024). In a human asthma lung tissue model, the YTHDF1 gene binds to the cyclin 1 (CCND1) mRNA modification site, thereby stimulating the proliferation and migration of airway smooth muscle cells, which are involved in airway spasm and contraction during asthma pathogenesis (Wang et al., 2023a). Meanwhile, in human and mouse airway epithelial cells, FTO knockout caused deletion of airway epithelial ciliated cells and exhibited asthmatic airway inflammatory manifestations with upregulation of IL-13 expression and downregulation of IL-12b expression, which may have been related to a modification in the expression of Foxj1 mRNA, a major regulator of motor ciliogenesis, by FTO-m^6^A (Kim et al., 2021). Leoni et al. identified enhanced mast cell responses to acute stimuli in mice knocked out of METTL3 and METTL14 genes, which may have been directly related to the fact that deletion of METTL3 and METTL14 increases mast cell effector center factors (TNF and IL-13) (Leoni et al., 2023). It is well known that the immune response occurring in mast cells mediated by immunoglobulin E is an effective stimulus for pathological changes in asthma (Kaliner, 1989). Deletion of YTHDF2 promotes demethylation of histone H3 lysine-27 trimethylation (H3K27me3) in response to bacterial-induced inflammation, which leads to enhanced production of pro-inflammatory cytokines and facilitates co-transcriptional deposition of m^6^A (Wu et al., 2020). In studies of lung tissue from asthmatic mice, H3K27me3 demethylation was found to regulate the contractile function and migratory reproduction function of airway smooth muscle in human asthma disease, as well as inhibit the expression of inflammatory factors IL-6 and IL-12B, which are involved in the regulation of the cellular inflammatory response as well as the development of asthma (Yu et al., 2018). METTL3 synergizes with YTHDF2 to promote PGC-1α mRNA degradation and affects monocyte inflammation and mitochondrial metabolism in a monocyte inflammation model (Zhang et al., 2021). In human and mouse asthma disease, PGC-1α expression was shown to be significantly upregulated in bronchial smooth muscle and was an important factor in the proliferation of asthmatic airway smooth muscle and impaired airway epithelial barrier dysfunction (Trian et al., 2007; Saito et al., 2021; Huang et al., 2023). In alveolar epithelial cells from mice with bronchopulmonary dysplasia, inhibition of YTHDC1 led to a decrease in IL-33 mRNA and protein expression, which in turn inhibited alveolar epithelial cell apoptosis and alleviated lung injury induced by hyperoxia treatment for bronchopulmonary dysplasia (Bao et al., 2023). Enrichment analysis and scoring against gene set library in the Kyoto Encyclopedia of Genes and Genomes database clarified that the genetic signature of IL-33 activation is elevated in neutrophilic and mixed granulocytic asthma (Badi et al., 2023).

In summary, m^6^A methylation regulates the release of certain asthma inflammatory factors, the contraction of airway smooth muscle cells, and the development of asthma subtypes, which are further mediated by regulatory factors such as YTHDF1, YTHDF2, YTHDC1, FTO, and METTL3, which have emerged as new targets for the treatment and prognosis of asthma.

## 3 The role of m^6^A RNA methylation in regulating inflammatory cells in asthma

### 3.1 m^6^A and mononuclear macrophages

m^6^A is involved in macrophage inflammatory response and polarization mediated by regulatory factors, and macrophages can be polarized into M1 and M2 macrophages. M1 macrophages promote the expression of Th1 and Th17 inflammatory cells and chemokines, and are involved in the development of NA and its steroid resistance, whereas M2 macrophages activate Th2 cells, promote the infiltration of lung eosinophils, and are involved in the development of EA (Saradna et al., 2018). Among them, the regulatory factors YTHDF1, YTHDF2, METTL3, METTL14, and FTO may play crucial roles in mediating m^6^A regulation of macrophage polarization and participate in the mechanisms underlying the formation of NA and EA subtypes.

#### 3.1.1 YTHDF1-m^6^A and mononuclear macrophages

YTHDF1 gene expression was upregulated in human macrophages infected by syphilis, whereas YTHDF1 knockdown upregulated the expression of M1 markers (CD86 and iNOS) and downregulated M2 markers (CD206 and ARG1); during the course of infection, m^6^A-YTHDF1 promoted the inhibitory factor of the important macrophage inflammatory pathway JAK2/STAT3-mRNA translation, thus negatively regulating macrophage-associated inflammatory responses and the secretion of the inflammatory factor IL-1β (Li et al., 2022). Blockade of IL-1β modulated Th17/Treg immune imbalance and attenuated neutrophil airway inflammation in an ovalbumin-induced mouse model of asthma (Chen et al., 2022). Studies in septicemic rats indicated that YTHDF1-m^6^A directly acts on the JAK2/STAT3 signaling pathway in macrophages, thereby inducing the upregulation of IL-17 expression, affecting Th1/Th17 balance and regulating inflammatory responses in macrophages (Xing et al., 2021). The JAK2/STAT3 signaling pathway in macrophages is an important target for allergic asthma development and is involved in important asthma pathological processes such as the activation of mast cells and the promotion of bronchial smooth muscle thickening and airway remodeling (Qu et al., 2022; Wang et al., 2022; Bai et al., 2023). Inhibition of the JAK/STAT pathway reduces neutrophil activation and improves the anti-inflammatory effects of corticosteroids in asthma patients (Milara et al., 2022). Considered together, m^6^A-YTHDF1 plays a complex role in the pathological process of inflammatory response in the NA asthma subtype through the regulation of the macrophage JAK2/STAT3 signaling pathway.

#### 3.1.2 YTHDF2-m^6^A and mononuclear macrophages

Yu et al. found for the first time that YTHDF2 expression was upregulated in lipopolysaccharide (LPS)-induced macrophage inflammatory response and regulated the inflammatory response through the YTHDF2-MAP2K4/MAP4K4 mRNA-p38 MAPK/p65 NF-κB/ERK-IL-6/IL-12/IL-1β pathway to regulate inflammatory response in macrophages (Yu et al., 2019). p38 MAPK, IL-6, and IL-1β are all important targets of the inflammatory response in NA asthma and are involved in the mechanism of steroid resistance in NA therapy (Sánchez-Ovando et al., 2021; Chen et al., 2022; Xie et al., 2022). In a mouse model of neutrophilic allergic airway inflammation, exogenous IL-25 ameliorated neutrophilia in the airway by inhibiting macrophage M1 polarization as well as IL-12 expression (Chang et al., 2023). Subsequently, the relationship between YTHDF2 and macrophage M1 and M2 polarization was first identified in a study of the macrophage polarization model. YTHDF2 induced M2 macrophage polarization by promoting the degradation of p53 mRNA, and inhibited M1 macrophage polarization by inhibiting the NF-κB, p38 and JNK signaling pathways (Cai et al., 2022). On the other hand, in mouse macrophages, YTHDF2 interacted with the RNA-binding motif 4 (RBM4) to promote YTHDF2-m^6^A degradation of STAT1 mRNA, which in turn inhibited IFN-r-, IL-6-, and IL-1β-induced macrophage M1 polarization but did not affect IL-4-induced M2 macrophage polarization (Huangfu et al., 2020). In summary, m^6^A-YTHDF2 inhibits M1 polarization and promotes M2 polarization by acting on signaling pathways such as NF-κB, MAPK, and STAT in macrophages, thereby playing an important role in the mechanism of asthma subtypes and targeted therapy.

#### 3.1.3 METTL3-m^6^A and mononuclear macrophages

METTL3-m^6^A suppresses the macrophage inflammatory response induced by LPS by inhibiting the NF-κB signaling factor to downregulate IL-6 and TNF-α expression (Wang et al., 2019). Another study of LPS-induced macrophages found that METTL3-m^6^A targeting of Irakm mRNA/TLR4 signaling in macrophages promoted macrophage activation (Tong et al., 2021) Recent m^6^A-seq sequencing of peripheral blood cells from children with allergic asthma revealed that METTL3 is lowly expressed in monocyte-derived macrophages from children with allergic asthma (Han et al., 2023). Further animal experiments confirmed that the above may be associated with the inhibition of M2 macrophage activation by METTL3-m^6^A through PI3K/AKT and JAK/STAT6 signaling, a process dependent on the degradation of PTX3 mRNA by METTL3-YTHDF3-m^6^A (Han et al., 2023). In pediatric pneumonia peripheral blood mononuclear cell and human embryonic lung cell models, downregulation of METTL3 modified the EZH2 gene, which in turn regulated the JAK2/STAT3 signaling pathway, inhibited the expression of IL-6 and TNF-α, and participated in the development of LPS-induced inflammatory response (Yang et al., 2021). CircN4bp1 mRNA may be upregulated in sepsis-induced acute respiratory distress syndrome macrophages with modification of METTL3-m^6^A, which further promotes M1 macrophage activation but inhibits M2 macrophage polarization through the CircN4bp1-miR-138–5p/EZH2 axis (Zhao et al., 2021). In a mouse model of asthma, EZH2 promoted asthma development through the FOXO3-miR-34b-BTG2 axis (Liu et al., 2021). In summary, the results of different studies on the role of METTL3-m^6^A in macrophage inflammation and activation are varied, which may be related to the multi-targets of METTL3-m^6^A and the complexity of the biological microenvironment, but it may be involved in macrophage differentiation and the development of asthma subtypes.

#### 3.1.4 METTL14-m^6^A and mononuclear macrophages

In macrophages from acutely bacterially infected mice, METTL14 was involved in macrophage activation through the METTL14/YTHDF1-SOCS1-TLR4-p65 NF-κB axis, which promoted the production of inflammatory factors TNF-α, IL-6, and inflammatory factor storm, and downregulated macrophage responses to acute bacterial infection in mice (Du et al., 2020). In a stroke model, depletion of METTL14 inhibited M1 macrophage polarization and promoted M1 to M2 conversion, which in turn attenuated brain injury (Li et al., 2023). In the inflammatory response of macrophages in atherosclerosis, m^6^A-METTL14 directly acted on the p65 NF-κB pathway to regulate IL-6 transcription, which in turn induced macrophage M2 polarization (Zheng et al., 2022), which is similar to the mechanism by which YTHDF2 mediates macrophage effects on the inflammatory subtype of asthma; therefore, it would be worthwhile to further investigate whether this mechanism is synergistically accomplished by both factors.

#### 3.1.5 FTO-m^6^A and mononuclear macrophages

Unlike other regulators, FTO promoted both M1 and M2 polarization in a mouse macrophage model, which may have been related to the fact that FTO promotes the phosphorylation levels of IKKα/β, IκBα, and p65 in the NF-κB signaling pathway while maintaining the mRNA stability of STAT1, STAT6, and PPAR-γ (Gu et al., 2020). During the macrophage inflammatory response in mice, m^6^A levels were expressed at higher levels in the harder microenvironment than in the rest of the microenvironment, and inhibition of the FTO gene promoted the expression of SOCS1 mRNA, a process that required YTHDF1-m^6^A mediation, while high expression of SOCS1 further inhibited the phosphorylation of STAT1 and p65 as well as the expression of inflammatory factors (IL-1β, IL-6), thereby regulating the inflammatory response and functional activation of macrophages in different tissue microenvironments (Hu et al., 2022). FTO targets macrophage polarization and inflammatory responses and may influence the mechanism of asthma subtype formation in different microenvironments.

#### 3.1.6 m^6^A and NLRP3-M2 macrophages

**FIGURE 4:**
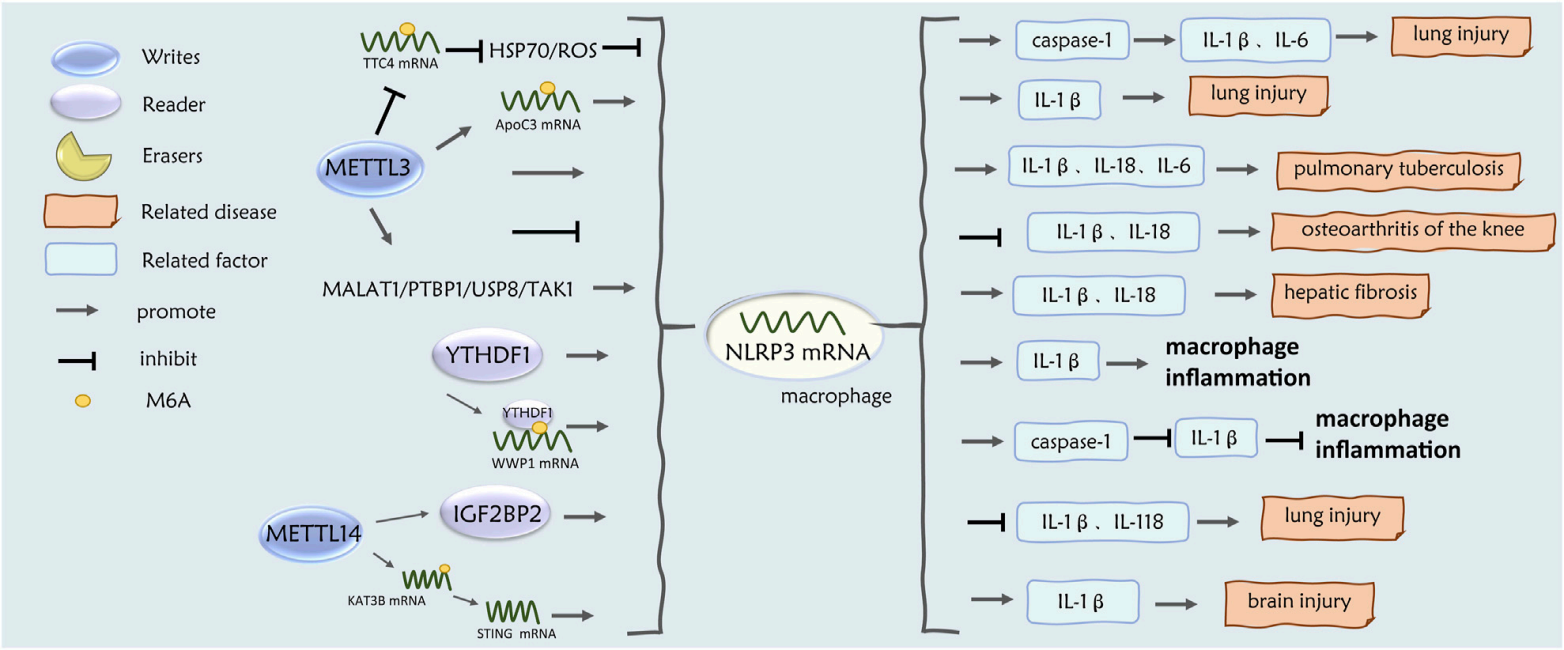
Role of m^6^A regulatory factors in NLRP3-M2 macrophages.

m^6^A in inflammatory response may be involved in macrophage polarization through modification of NLRP3 mRNA in regulating inflammatory response and inflammatory subtypes. Liu et al. found that NLPR3 promotes M2 macrophage polarization and inhibits inflammatory response under the regulation of ubiquitin-specific protease 19 and autophagy in a series of animal and cellular experiments (Liu et al., 2021). On the one hand, M2 macrophages promote Th2 cell activation in asthma, and on the other hand, NLRP3 is an important transcription factor for Th2 cell differentiation and induces Th2 inflammatory responses in asthmatic mice (Bruchard et al., 2015). However, inhibition of NLRP3 inflammatory vesicles attenuated neutrophil airway inflammation in asthmatic neutrophil airway inflammation in a mouse model of neutrophil airway inflammation and in human asthmatics (Liu et al., 2023). The roles of METTL14, METTL3, and YTHDF1 in NLRP3 and macrophage polarization and macrophage inflammation are described below (Figure 4).

Animal studies revealed that METTL14 levels were upregulated in macrophages from ischemic stroke rats, and KAT3B expression was enhanced by m^6^A modification of KAT3B mRNA, whereas knockdown of the METTL14 gene promoted macrophage switching from M1 to M2 via KAT3B-STING signaling and further inhibited NLRP3 inflammatory vesicles in macrophages to decrease IL-1β levels and alleviate brain injury (Li et al., 2023). Recent studies have shown that METTL14 was significantly upregulated in macrophages in a mouse model of lung injury (ALI), and that METTL14-m^6^A modified the NLRP3 transcript, which was subsequently recognized by IGF2BP2 to stabilize the expression of NLRP3 mRNA, thereby promoting the secretion of IL-1β and IL-18, leading to lung injury (Cao et al., 2024).

In related studies, it was found that in the ALI mouse model, METTL3-m^6^A mediated the expression of apolipoprotein C3 (ApoC3) mRNA in macrophages and promoted the activation of NLRP3 inflammatory vesicles, which led to macrophage pyroptosis and the release of inflammatory factors such as IL-1β, thus exacerbating the lung injury (Pu et al., 2024). On the other hand, METTL3-m^6^A inhibited the stability of the TTC4 gene, further inhibiting the mitochondrial damage occurring in the HSP70/ROS/NLRP3/Caspase-1 signaling pathway, and reduced the levels of inflammatory factors such as IL-1β, IL-6, thus reducing the inflammation and scorched death of macrophages in the ALI mouse model (Chen et al., 2023). Animal studies confirmed that IL-18 may initiate asthma-induced eosinophil production and airway obstruction (Mishra et al., 2022). In a mouse model of knee osteoarthritis, extracellular vesicles inhibited the downregulation of NLRP3 expression activity via modification of NLRP3 mRNA by METTL3-m^6^A in macrophages and inhibited the secretion of IL-1β and IL-18 inflammatory factors and inflammatory responses (Zhou et al., 2022). Recent studies have confirmed that METTL3-MA modification promotes *Mycobacterium tuberculosis*-induced NLRP3 activation and inflammatory responses in mouse macrophages and promotes the secretion of inflammatory factors such as IL-1β, IL-18, IL-6, and TNF-α (Han et al., 2024).

Additional *in vivo* and *in vitro* experimental studies have shown that TYHDF1 is upregulated under sepsis environmental conditions and mediates m^6^A modification of WWP1 mRNA in macrophage cell line RAW264.7 cells, which promotes the translation of WWP1 and consequently inhibits apoptosis and the release of the IL-1β inflammatory factor via the WWP1/NLRP3/caspase-1 axis (Zhang S. et al., 2022). In another study in septic mice, YTHDF1-m^6^A methylation directly promoted the translation of NLRP3 mRNA and induced IL-1β production in macrophages (Hao et al., 2022). In bacterially infected mouse macrophages, YTHDF1 promoted NLRP3 expression in macrophages and IL-1β production during bacterial infection (Hao et al., 2022).

m^6^A methylation acts as an upstream signal to promote or inhibit the activity of NLRP3 inflammatory vesicles, which regulates M2 polarization and the secretion of inflammatory factors in macrophages; therefore, further study of m^6^A-NLRP3-macrophage-asthma is of great significance in providing precisely targeted therapy to EA and NA.

In summary, m^6^A exerts its methylation function with the assistance of the above regulatory factors to regulate M1 and M2 polarization and inflammatory responses in macrophages, thereby affecting the development of asthma subtypes. Although the conclusions we obtained seem to be contradictory, which may be related to the cellular microenvironment and hardness, further study is needed to clarify the specific mechanism.

### 3.2 m^6^A and T lymphocytes

m^6^A is closely related to the differentiation and activity function of T cell subsets, and the type of inflammatory response induced can be categorized into Th1, Th17, and Th2 inflammatory responses based on T cell subsets (Luo et al., 2022), such as Th2 regulates EA (Borish, 2016). Th17 and Th1 coordinate neutrophil recruitment to the lungs, induce steroid resistance in NA asthma, and are the main inflammatory cells involved in the development of the NA subtype (Sánchez-Ovando et al., 2021; Luo et al., 2022).

#### 3.2.1 METTL3-m^6^A and T lymphocytes

METTL3 was shown to be highly expressed in CD4^+^ T cells obtained from the peripheral blood of asthma patients, which may have been due to the fact that METTL3 synergizes with YTHDF2 to mediate a reduction of Foxp3 mRNA stability by m^6^A, leading to dysregulation of Treg/Th17 homeostasis and regulating inflammatory responses in asthma (Fan et al., 2022). Animal studies have shown that deletion of the METTL3 gene in T cells affects the differentiation and maturation of naïve T cells, thus leading to a decrease in the production of Th1 and Th17 cells and an increase in Th2 cells, mainly due to the fact that METTL3-m^6^A has the ability to regulate the balance of the two basic signaling pathways of T cell homeostasis (IL-17/JAK-STAT and TCR/ERK/AKT) (Li et al., 2017). Recent studies have shown that low METTL3 expression in the peripheral blood of Th2 asthma patients is correlated with disease severity, and further animal experiments confirmed that decreasing METTL3-m^6^A methylation activity regulated CD4^+^ T cell activity by increasing the stability of SOX5 mRNA in bronchial epithelial cells, which in turn upregulated GATA3/Th2 activity, inhibited T-bet/Th1 activity, and promoted the development of Th2 asthma (Chen et al., 2024). In an autoimmune encephalomyelitis model, METTL3 maintained Th17 differentiation and activity by targeting and modifying the stability of SOCS1 and SOCS3 mRNAs in Th17 cells (Wang et al., 2023). In T cells sampled from patients with autoimmune uveitis (EAU), METTL3, assisted by YTHDC2 was targeted to inhibit Th17 function and inflammatory responses via the m^6^A-ASH1L mRNA- IL-17A/17F/23R-Th17 pathway (Zhao et al., 2023). In acute infection models, deletion of METTL3 in T cells has been shown to impair T-bet expression and inhibit Th1 cell differentiation (Yao et al., 2021). In contrast, studies of immune rejection revealed that METTL3 deficiency inhibited T-bet expression and impaired Th1 cell differentiation in allogeneic T cell responses (Li et al., 2022). T-bet is a key transcription factor required for Th1 cell differentiation and is inextricably linked to NA development (Kallies and Good-Jacobson, 2017). METTL3-m^6^A regulates Th1 and Th17 differentiation and activity, and its role in NA asthma inflammatory subtypes should be explored in depth.

#### 3.2.2 Other regulators-m^6^A and T lymphocytes

Knockout of the FTO gene in mouse lung tissue cells resulted in significant asthma-like inflammation in mice, as evidenced by upregulation of Th2 cell marker IL13 mRNA levels and downregulation of Th1 cell marker IL12b and the T cell antibody CD8b1 (Kim et al., 2021). In contrast, in a mouse model of spontaneous colitis, mouse METTL14 gene deficiency caused upregulation of Th1 and Th17 cells (Lu et al., 2020). In the EAU model, deletion of ALKBH5 in T cells inhibited the IL-17 signaling pathway and IFN-γ secretion in CD4^+^ T cells, while specifically suppressing the ability of T cells to recruit neutrophils to the central nervous system during neuroinflammation (Zhou et al., 2021). FTO, ALKBH5, and METTL14 are thought to regulate T-cell differentiation and inflammatory response, thus having a potential role in the mechanism of different subtypes of asthma disease.

Under the multiple modes of action of regulatory factors, m^6^A plays a complex regulatory role in the differentiation and function of Th cell subpopulations, and in-depth studies are needed regarding its role in regulating the balance of Th2/Th17 and thus its involvement in the diagnosis and management of asthma subtypes.

### 3.3 m^6^A and neutrophils

#### 3.3.1 m^6^A and neutrophil infiltration

Th17- and Th1-induced non-Th2 inflammatory responses drive the development of NA, which is mainly characterized by sputum-induced neutrophilia and infiltration of inflammatory factors such as IL-1β, IL-6, IL-12, and IL-17. In LPS-induced inflammation in mice, METTL3-m^6^A modified TLR4 mRNA to induce TLR4 protein expression in neutrophils, which further enhanced the migration of bone marrow neutrophils into the bloodstream via TLR4/CXCL2 and promoted TNF-α, IL-6, and IL-1β inflammatory factors in neutrophils via TLR4/NF-κB signaling pathway production. (Luo et al., 2023). ALKBH5 expression was downregulated in neutrophils sampled from septicemia mice, and ALKBH5-m^6^A methylation maintained the intrinsic ability of neutrophils to undergo chemotaxis toward the site of infection, ensuring their antimicrobial and immune functions, due to the function of ALKBH5-m^6^A in maintaining CXCR2 mRNA stability (Liu et al., 2022). Inhibition of the chemokine CXCR2 is involved in peripheral neutrophil migration and ameliorates neutrophil-dependent airway inflammation in asthmatics and asthmatic mice (Mattos et al., 2020; Zhu et al., 2020).

#### 3.3.2 m^6^A and NETs

The process by which neutrophils release elastase, histone G, myeloperoxidase, and DNA in response to infection is known as NETs, which creates anti-infective effects in the organism and can cause tissue damage (Lee and Grinstein, 2004; Silva et al., 2021). The release of NETs is a key upstream signal that induces the onset of NA and significantly correlates with the severity of asthma development (Wan et al., 2020; Janssen et al., 2022). NETs may target m^6^A to regulate iron death and autophagy, thus participating in NA.

PPARγ inhibits Th17 responses by modulating reactive oxygen species (ROS) signaling in an LPS-induced asthma mouse model (Miao et al., 2021). GPX4-induced iron death occurs to exacerbate the progression of asthma disease in humans, and inhibition of GPX4 gene expression attenuates bronchial epithelial cell damage and NA progression in mice (Bao et al., 2022; Nagasaki et al., 2022). In a mouse model of sepsis, NETs induced iron death in alveolar epithelial cells through activation of METTL3-m^6^A modification, a pathway that contributes to lung injury and systemic inflammation by promoting iron death via the NETs/p300/H3K27ac/METTL3-m^6^A/IGF2BP2-m^6^A/HIF-1α/GPX4/ROS pathway (Zhang et al., 2023). In a mouse model of sepsis-associated acute lung injury (SI-ALI), NETs induced METTL3-m^6^A modification of GPX4 mRNA, which inhibited GPX4 expression by reducing GPX4 mRNA stability and promoted iron death in alveolar epithelial cells, while NETs specifically exacerbated iron death through the TLR9/MyD88/NF-kβ/METTL3-m^6^A/GPX4/ROS pathway to exacerbate iron death and contribute to the development of lung injury (Zhang et al., 2022). In a mouse model of acute kidney injury, knockdown of METTL3 reduced the downregulation of GPX4 expression and upregulation of ROS expression in LPS-treated HK-2 cells, promoting iron death (Hu et al., 2023). Considered together, m^6^A methylation may be involved in the mechanism of NA asthma by inducing the onset of iron death via GPX4/ROS under the NETs activation.

In asthmatics, increased autophagy plays a role in promoting Th2-type immune response and eosinophilic inflammation, whereas decreased autophagy plays an important role in NA (Barnes et al., 2022). In a mouse model of SI-ALI, NETs contribute to alveolar epithelial cell injury by causing impaired autophagic flux through the METTL3-m^6^A/Sirt1 mRNA pathway (Qu et al., 2022). In a mouse model of osteoarthritis, METTL3 mediates m^6^A regulation of autophagy-associated 7 (ATG7) mRNA stability to impair autophagic flux in a YTHDF2-dependent manner (Chen et al., 2022). In animal experiments, METTL3 silencing enhanced autophagic flux and inhibited apoptosis in ischemia-reperfusion-treated cardiomyocytes (Song et al., 2019). Considered together, the process of m^6^A methylation targeting NETs to promote NA may be related to autophagy.

Accordingly, we hypothesized that m^6^A could serve as an intermediate target for NETs-induced asthma development, which is involved in neutrophil aggregation in asthma disease and is an important target for the mechanism underlying NA subtype development.

### 3.4 m^6^A and eosinophils

Infiltration of eosinophils in sputum induction is an important criterion for the diagnosis of EA (Simpson et al., 2006; Komlósi et al., 2022). According to the data analysis conducted by Sun et al., there was a significant correlation between YTHDF3 and EIF3B and the infiltration of eosinophils in severe asthma, with the expression of YTHDF3 being positively correlated and the expression of EIF3B being negatively correlated (Sun et al., 2021). However, there have been no laboratory studies conducted on the specific mechanisms of YTHDF3 and EIF3B with eosinophils and EA. However, recent studies in mice with allergic rhinitis found that METTL3-m^6^A methylation enhances circMIRLET7BHG expression, and circMIRLET7BHG in mouse epithelial cells contributes to OVA-induced allergic symptoms by modulating inflammatory responses such as eosinophil infiltration (Zhan et al., 2023).

m^6^A methylation is unequivocally associated with eosinophil infiltration and the development of EA in asthma; however, more detailed studies on the specific mechanisms and pathological processes of m^6^A-induced EA development are needed by researchers.

### 3.5 m^6^A and NK cells

NK cells are one of the inflammatory bases of asthma disease development (Komlósi et al., 2022). An animal study showed that YTHDF2, which is upregulated in NK cells that are activated by cytokines or viral infection, maintained NK cell homeostasis and terminal maturation, regulated NK cell trafficking, and promoted the intrinsic function of NK cells through the formation of a STAT5-YTHDF2–positive feedback loop (Ma et al., 2021). In mouse spleen FTO-deficient NK cells, the expressions of IL-2, IL-15, and the JAK/STAT signaling pathway were significantly elevated, mainly due to the fact that FTO regulates the stability of SOCS3 mRNA through demethylation to modulate NK cell homeostasis and antitumor immunity (Kim et al., 2023). IL-2 inhibited the expression of IL-17A in TLR9-induced asthmatic mice (Murakami et al., 2020). Further studies are needed to demonstrate the mechanisms linking m^6^A and NK cells in asthma, which would lead to the discovery of new strategies for asthma treatment.

### 3.6 m^6^A and dendritic cells

m^6^A plays a complex role in the activation and function of DC cells, which are involved in asthma inflammation. DC cell apoptosis and activation are essential for maintaining the Th2/Th17 balance in asthma. Depending on the degree of their maturation and route of action, DCs can play different roles in inflammatory progression in NA and EA (Park et al., 2020; Izumi et al., 2021; Deng et al., 2022).

Animal studies revealed that the reduced levels of asthma-associated inflammatory factors IL-6 and IL-12 mRNA in DC cells knocked down for METTL3 may have been related to a defective TLR4/NF-κB signaling pathway caused by the deletion of METTL3. In addition, METTL3-m^6^A upregulated the translation of key DC transcripts (CD40, CD80, and TLR signaling junctions) to promote DC maturation and activation (Wang et al., 2019a). Animal studies revealed that m^6^A modified lncRNA lnc-Dpf3 demethylation and upregulated lnc-Dpf3 interacting with the transcription factor hypoxia-inducible factor 1-alpha (HIF-1alpha), which suppressed the migratory capacity of DCs and inhibited the expression of inflammatory factors associated with IFN-γ and IL-17 in Th1 and Th17 cells (Liu et al., 2019).

The role of m^6^A methylation in the function of DC cells is contradictory, but the role of m^6^A-targeted DCs in regulating Th2/Th17 homeostasis is important for the progression of inflammatory subtypes in asthma and remains to be investigated.

## 4 m^6^A RNA methylation in asthma treatment

Screening of the GEO database identified six m^6^A-related genes (FTO, IGF2BP2, RBM15, RBMX, WTAP, and YTHDC1) that are significantly dysregulated in asthma disease, and further using the CMap database, we found that resveratrol may target these dysregulated m^6^A genes and could serve as a potential therapeutic agent for asthma (Mo et al., 2022).

### 4.1 m^6^A and glucocorticoids

Glucocorticoids are the most commonly used drugs for the treatment of asthma, although steroid resistance is a challenge in the NA subtype, which reduces the therapeutic effect. After nasal mucosal drug sensitivity analysis, methylprednisolone was found to be closely related to METTL14 expression, and topical nasal methylprednisolone can potentially improve asthma respiratory allergy symptoms by regulating METTL14 expression (Wang et al., 2023). Inhibition of ALKBH5-m^6^A modification ameliorates glucocorticoid resistance in T-cell acute lymphoblastic leukemia, which may be related to modification of the resistance gene USP1 by ALKBH5-m^6^A (Gong et al., 2021). In a mouse model of nonalcoholic fatty liver disease, FTO knockdown significantly attenuated dexamethasone-induced fatty liver in mice, which may be related to glucocorticoid receptor–mediated FTO trans-activation and m^6^A demethylation (Hu et al., 2020). These results indicate the potential mechanism of action of m^6^A on glucocorticoids, which establishes an important direction for addressing steroid resistance in asthma.

### 4.2 m^6^A and T-bet

In animal experiments, inhibition of METTL3 in CD4^+^ T cells of STM2457-treated mice mediated downregulation of m^6^A levels, which in turn suppressed T-bet expression and inhibited Th1 differentiation of CD4^+^ T cells (Li et al., 2022). In a mouse model of viral infection, METTL3-m^6^A regulated T-bet expression to regulate the CD8^+^ T cell effector differentiation function (Guo et al., 2023). In a mouse model of asthma, dexamethasone and hemp mustard calming barb (*Majie cataplasm*) modulated the Th1/Th2 balance of asthmatic mice by regulating T-bet, which was significantly reduced in dexamethasone-treated asthmatic mice and significantly increased in *M. cataplasm*-treated asthmatic mice (Ji et al., 2020). In an OVA-induced asthma mouse model, locust yellow regulated Th1/Th2 and Treg/Th17 homeostasis by acting on the Th1 transcription factor T-bet (Liang et al., 2017). Thus, m^6^A may precisely treat asthma inflammatory subtypes by targeting T-bet transcription factors.

### 4.3 m^6^A and TLR4/NF-KB

m^6^A can participate in the inflammatory response of asthma-associated inflammatory cells through the TLR4/NF-ΚB pathway. For example, METTL14/YTHDF1-m^6^A is involved in macrophage activation and the release of inflammatory factor IL-6 via TLR4/NF-ΚB in bacteria-infected mice (Du et al., 2020). In animal experiments, METTL3-m^6^A promoted IL-6, IL-1β production in neutrophils as well as reduced IL-6 and IL-12 levels in DC cells through the TLR4/NF-κB signaling pathway (Wang et al., 2019a; Luo et al., 2023). Inhibition of the TLR4/NF-ΚB pathway by FTO-m^6^A in turn inhibited TNFα and IL6 expression in the microglia in a mouse model of EAU (He et al., 2023). Meanwhile, Dubey et al. discovered that in cardiomyocytes from LPS-induced endotoxemia mice, FTO expression downregulated while increasing the overall m6A-RNA methylation level, which promoted the expression of IL-6, IL-1β, and TNF-α proinflammatory factors that aggravated myocardial injury (Dubey et al., 2022).

In a rat asthma model, Yanghe Pingchuan granules, traditional Chinese medicine, alleviated bronchial asthma airway inflammation by blocking the TLR4/NF-κB/NRLP3 signaling pathway to reduce the levels of inflammatory factors IL-1β and IL-18 in rat bronchial tissues (Pan et al., 2022). In addition, in a mouse model of OVA/LPS-induced neutrophilic airway inflammation in asthma, MUC1-CT attenuated neutrophilic airway inflammation in asthma and resisted steroid resistance through inhibition of the TLR4/NF-κB pathway (Liu et al., 2023). Dexmedetomidine (DEX) is a highly selective α2 adrenergic receptor agonist, which may ameliorate the Th2 inflammatory response in asthmatic mice by inhibiting TLR4/NF-κB (Zhou et al., 2023). Another related study confirmed that DEX attenuates asthma airway hyperresponsiveness and airway inflammation by reducing Th2-type cytokine production through inhibition of TLR4/NF-κB signaling in an OVA-induced asthma model in mice.

Considered together, m^6^A may pave the way for the treatment of inflammatory subtypes of asthma disease by modulating TLR4/NF-ΚB-related pathways, which may become a new therapeutic approach.

### 4.4 m^6^A and vitamin D3

In a human cytomegalovirus infection model, vitamin D3 inhibited METTL3-m^6^A-mediated apoptosis by downregulating METTL3 through inhibiting AMPK activation (Zhu et al., 2022). Vitamin D_3_ (VitD3) is important in the adjunctive treatment of asthma, and supplementation with VitD3 induced an enhanced blockade of the antibody response and suppressed the eosinophilic inflammatory response as well as the expression of IL-10 in lung tissues (Hesse et al., 2020). *In vitro* experiments demonstrated that in lung fibroblasts, VitD3 may increase the efficacy of beclomethasone 17-propionate and montelukast sodium in resisting asthmatic airway remodeling at the mRNA level (Sobczak and Pawliczak, 2022).

### 4.5 m^6^A and PGC-1α

In oxLDL-induced THP1 monocyte inflammation, METTL3/YTHDF2-m^6^A promotes the degradation of PGC-1α mRNA and facilitates monocyte inflammation and mitochondrial metabolism (Zhang et al., 2021). In a rat model of insulin resistance, METTL14 and YTHDF2 may coordinately mediate m^6^A methylation to regulate PGC-1α mRNA translation and stability, which induces iron death and insulin resistance (Zhang et al., 2023). PGC-1α mRNA is a key target in the mechanism of asthma, and in a mouse model of asthma airway smooth muscle cell proliferation, icariin increased activation of the SIRT1/AMPK/PGC-1α axis by inhibiting the expression of miR-138-5, ameliorating asthma oxidative stress and airway remodeling (Huang et al., 2023). Additional *in vitro* experiments have demonstrated that montelukast and zafirlukast enhance mitochondrial biogenesis and strengthen mitochondrial function by promoting the expression of PGC-1α mRNA in human bronchial epithelial cells, which is a novel pharmacological route for the treatment of asthma (Wang et al., 2019c; Ren et al., 2021).

In conclusion, m^6^A is a new target for the treatment of asthma, which is expected to address the role of NA steroid resistance and precisely target NA and EA for the treatment of asthma disease.

## 5 The role of m^6^A in asthma prognosis

Su et al. (Sun et al., 2021) used 87 healthy controls and 344 severe asthma cases from the Unbiased Biomarkers for Predicting Outcomes of Respiratory Diseases (U-BIOPRED) study to systematically assess the pattern of m^6^A modifications mediated by 27 m^6^A modifiers, as well as the impact of their immune microenvironmental characteristics, by using the Wilcox test, R language for the 27 modifiers, logistic regression, and bioinformatics analysis such as COX regression analysis, which led to the discovery that YTHDF3 and YTHDC1 regulators play a crucial roles in severe asthma and can influence the prognosis of severe asthma. Another similar study extensively included RNA-modified regulatory factors and found that m^6^A RNA methylation of writer WTAP is closely associated with the development of severe asthma (Lin et al., 2022). Nutritional status largely influences the severity and prognosis of asthma. A recent META regression analysis study suggested that leptin levels were significantly higher in severe asthma cases than in mild asthma cases in the overall population and in Asians, independent of age and the sex ratio of the overall population, and may be a risk predictor and prognostic marker of asthma, which may be related to the association of leptin with obesity and body mass index in asthmatics (Wang et al., 2023d). Subsequent animal studies indicated that in an obese mouse model, FTO acted on CX3Cl1 mRNA to promote its de-m^6^A methylation, enhance its stability and half-life, and further participate in the hypothalamic leptin resistance function through the upregulation of CX3CL1/CX3CR1/SOCS3 (Liu et al., 2024). A multicenter cohort study confirmed the role of TGF-β in severe asthma and that its plasma levels were not affected by oral steroid medications (Sparreman et al., 2022). Another cellular assay confirmed that TGF-β can be an important factor for inducing epithelial-mesenchymal transition in human lung epithelial cells, which promotes the development of severe asthma and airway remodeling, greatly affecting the prognosis of asthma disease (Cheng et al., 2022). Another animal study indicated that in LPS-induced mouse Kupffer cells, METTL3/METTL14-m^6^A modification of TGF-β1 mRNA promoted the expressions of TGF-β1, IL-1β, and IL-6 as well as the development of hepatic fibrosis (Feng et al., 2021). Considered together, the m^6^A regulators YTHDF3, YTHDC1, WTAP, FTO, METTL3, and ETTL14 may have a significant effect on the prognosis of asthma, but studies on m^6^A methylation, severe asthma, and the prognosis of asthma are still in the initial stage and require further exploration by researchers.

## 6 Conclusion

m^6^A methylation plays an important role in the transcription and translation of many genes in the body in the presence of its different regulators, through which it becomes involved in the pathogenesis of asthma through a wide range of pathways, thereby affecting the inflammatory subtypes of asthma and steroid resistance during asthma treatment (Yang et al., 2018; Sun et al., 2021). Under the action of different cellular microenvironments and regulatory factors, m^6^A may produce multiple different or even contradictory effects and participate in the mechanisms of the development of inflammatory subtypes of asthma. Here, we discuss how m^6^A regulatory factors regulate the pathogenesis and treatment of different subtypes (EA, NA) of asthma disease, which may be of great value as important biomarkers for the prognosis and treatment of asthma subtypes.

Although we have gained a deeper understanding of asthma subtypes based on m^6^A-related mechanisms, the current study still has some limitations. First, the complex roles that m^6^A regulators play in different inflammatory cells make it difficult to clearly elucidate how they interact with each other to regulate the pathologic process of asthma subtypes, which requires us to understand their indispensable roles from a higher perspective. In addition, although there have been some studies on m^6^A-related combination drugs to improve asthma symptoms and steroid resistance, most of them remain at the mechanistic level, and clinical applications need to be further investigated.

m^6^A is a new way to diagnose asthma subtype classification and personalize treatment based on the subtypes of asthma. Different subtypes of asthma are regulated by different m^6^A-related genes, so exploration of these genes will guide the development of precise targeted therapies. The complex relationship between m^6^A regulators, asthma subtypes, and the specific mechanisms involved is an important direction for the researchers in the future.
